# Neuronal Calcium Sensor-1 Protects Cortical Neurons from Hyperexcitation and Ca^2+^ Overload during Ischemia by Protecting the Population of GABAergic Neurons

**DOI:** 10.3390/ijms232415675

**Published:** 2022-12-10

**Authors:** Elena G. Varlamova, Egor Y. Plotnikov, Egor A. Turovsky

**Affiliations:** 1 Institute of Cell Biophysics of the Russian Academy of Sciences, Federal Research Center “Pushchino Scientific Center for Biological Research of the Russian Academy of Sciences”, 142290 Pushchino, Russia; 2 A.N. Belozersky Institute of Physico-Chemical Biology, Lomonosov Moscow State University, 119992 Moscow, Russia; 3 V.I. Kulakov National Medical Research Center of Obstetrics, Gynecology and Perinatology, 117997 Moscow, Russia

**Keywords:** neuronal calcium sensor-1, oxygen-glucose deprivation, cell death, cortex, neurons, neuroprotection, signaling, calcium, gene expression, GABA, apoptosis, necrosis

## Abstract

A defection of blood circulation in the brain leads to ischemia, damage, and the death of nerve cells. It is known that individual populations of GABAergic neurons are the least resistant to the damaging factors of ischemia and therefore they die first of all, which leads to impaired inhibition in neuronal networks. To date, the neuroprotective properties of a number of calcium-binding proteins (calbindin, calretinin, and parvalbumin), which are markers of GABAergic neurons, are known. Neuronal calcium sensor-1 (NCS-1) is a signaling protein that is expressed in all types of neurons and is involved in the regulation of neurotransmission. The role of NCS-1 in the protection of neurons and especially their individual populations from ischemia and hyperexcitation has not been practically studied. In this work, using the methods of fluorescence microscopy, vitality tests, immunocytochemistry, and PCR analysis, the molecular mechanisms of the protective action of NCS-1 in ischemia/reoxygenation and hyperammonemia were established. Since NCS-1 is most expressed in GABAergic neurons, the knockdown of this protein with siRNA led to the most pronounced consequences in GABAergic neurons. The knockdown of NCS-1 (NCS-1-KD) suppressed the basic expression of protective proteins without significantly reducing cell viability. However, ischemia-like conditions (oxygen-glucose deprivation, OGD) and subsequent 24-h reoxygenation led to a more massive activation of apoptosis and necrosis in neurons with NCS-1-KD, compared to control cells. The mass death of NCS-1-KD cells during OGD and hyperammonemia has been associated with the induction of a more pronounced network hyperexcitation symptom, especially in the population of GABAergic neurons, leading to a global increase in cytosolic calcium ([Ca^2+^]_i_). The symptom of hyperexcitation of neurons with NCS-1-KD correlated with a decrease in the level of expression of the calcium-binding protein-parvalbumin. This was accompanied by an increase in the expression of excitatory ionotropic glutamate receptors, N-methyl-D-aspartate and α-amino-3-hydroxy-5-methyl-4-isoxazolepropionic acid receptors (NMDAR and AMPAR) against the background of suppression of the expression of glutamate decarboxylase (synthesis of γ-aminobutyric acid).

## 1. Introduction

The separate populations of GABAergic neurons are more sensitive to conditions of oxygen and glucose deficiency. It is known that with ischemia lasting 40 min or more, up to 80% of hippocampal cells in culture are damaged and killed. First of all, astrocytes and GABAergic neurons are damaged, as a result of which the neuroglial network is disrupted, which leads to the massive death of glutamatergic neurons as well [1,2]. The death of nerve cells is characterized by a rapid disruption of the mechanisms of ATP production and the maintenance of Ca^2+^ homeostasis of nerve cells and, subsequently, the activation of membrane-destroying enzymes [3]. It is known that various mechanisms of transport and compartmentalization function in the cell to maintain Ca^2+^ homeostasis [4]. In addition, the mechanisms of calcium binding by calcium-binding proteins (CaBP) are also active [5]. Calcium-binding proteins mediate various spatiotemporal patterns of intracellular Ca^2+^ signaling and interaction with numerous target proteins via cytosolic Ca^2+^ binding [6].

Neuronal calcium sensor-1 (NCS-1) was first discovered in the Drosophila nervous system [7] and has since been identified in many classes of organisms, from yeast to humans. NCS-1 is expressed in endocrine, heart, smooth muscle, and gastrointestinal tract tissue. In the brain, NCS-1 is most expressed in the cortex, but is also found at high levels in the hippocampus and dorsal root ganglion cells [8,9,10]. In the human genome, NCS proteins are encoded by 14 genes; however, amino acid sequences are highly conserved and can also be found in unicellular microorganisms [11]. Almost all members of the NCS family contain 2–3 functional Ca^2+^ binding domains, as well as an N-terminal myristoylation site [12]. NCS-1 regulates many different cellular functions, including exocytosis, neurite outgrowth, neuroprotection, axonal regeneration, nuclear Ca^2+^ regulation, and abnormalities in NCS-1 expression and activity contribute to a number of diseases [12,13,14,15]. NCS-1 is known to regulates many aspects of neuronal plasticity, such as short-term plasticity [16], long-term potentiation [17] and long-term depression [18], and facilitates rapid-acquisition of spatial memory [9]. NCS-1 is involved in the genesis of a number of neurodegenerative diseases—in sleep-wake disturbance symptoms in bipolar disease, autism, Parkinson’s disease, and is also involved in the induction of cocaine addiction [19,20,21,22].

Thus, the lack of knowledge of the molecular mechanisms of the regulation of the processes of neurotransmission by the NCS-1 protein, the survival of neurons, and the genesis of neurodegenerative diseases, necessitates their study.

## 2. Results

### 2.1. Different Expression of NCS-1 in the Cortical Cells in Culture In Vitro

**Figure 1:**
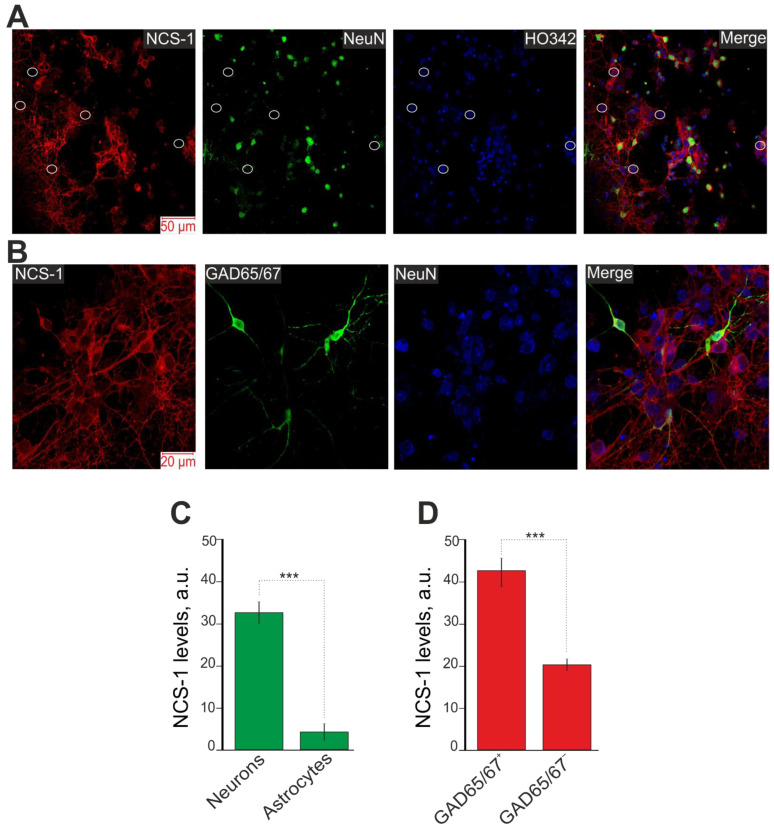
Immunocytochemical staining of mouse cortex cell culture with antibodies against NCS-1 and NeuN (**A**) and NCS-1, GAD65/67 and NeuN (**B**). Cell nuclei for panel (**A**) were stained with Hoechst 33,342 (HO342). Merge—the overlay of antibody fluorescence. For panel (**A**), white circles indicate astrocytes (cells without NeuN fluorescence). (**C**,**D**) Intensity levels of NCS-1 expression were determined by confocal imaging. For panel (**C**), we analyzed the fluorescence of secondary antibodies to NCS-1 in cells with fluorescence of secondary antibodies to GAD65/67 (neuronal marker) and without fluorescence of NeuN (astrocytes). For panel (**D**), we analyzed the fluorescence of secondary antibodies to NCS-1 in cells with fluorescence of secondary antibodies to GAD65/67 (GABAergic neurons) and without fluorescence of GAD65/67 (GAD65/67^−^, non-GABAergic neurons). The quantative data reflecting the level of NCS-1 expression are presented as fluorescence intensity values in summary bar charts (mean ± SEM). The values were averaged by 150 neurons for each column. We used the scans from three independent view fields for each experimental group. Statistical significance was assessed using paired *t*-test, *** *p* < 0.001.

The cell cultures that we used for experiments include two types of cells—neurons (71 ± 26%) and astrocytes (14 ± 12%) (Supplementary Figure S1). The cell cultures do not contain other cell types, since the cultivation of microglia requires the addition of IL-34, TGF-β and ovine wool cholesterol to the culture medium. Immunocytochemical staining of a cell culture of the mouse cerebral cortex with antibodies against NCS-1 and NeuN showed that in neurons (NeuN) the level of NCS-1 expression is more than three times higher than in astrocytes (Figure 1A, indicated by circles; Figure 1C). Neurons in the cell culture of the cerebral cortex are divided into two types according to their ergicity—GABAergic and glutamatergic. A marker of GABAergic neurons is cell staining with antibodies against glutamate decarboxylase (GAD65/67^+^), and neurons not stained with these antibodies (GAD65/67^−^). It turned out that in GAD65/67^+^-neurons the level of NCS-1 is two times higher than in GAD54/67 neurons (Figure 1B,D).

**Figure 2:**
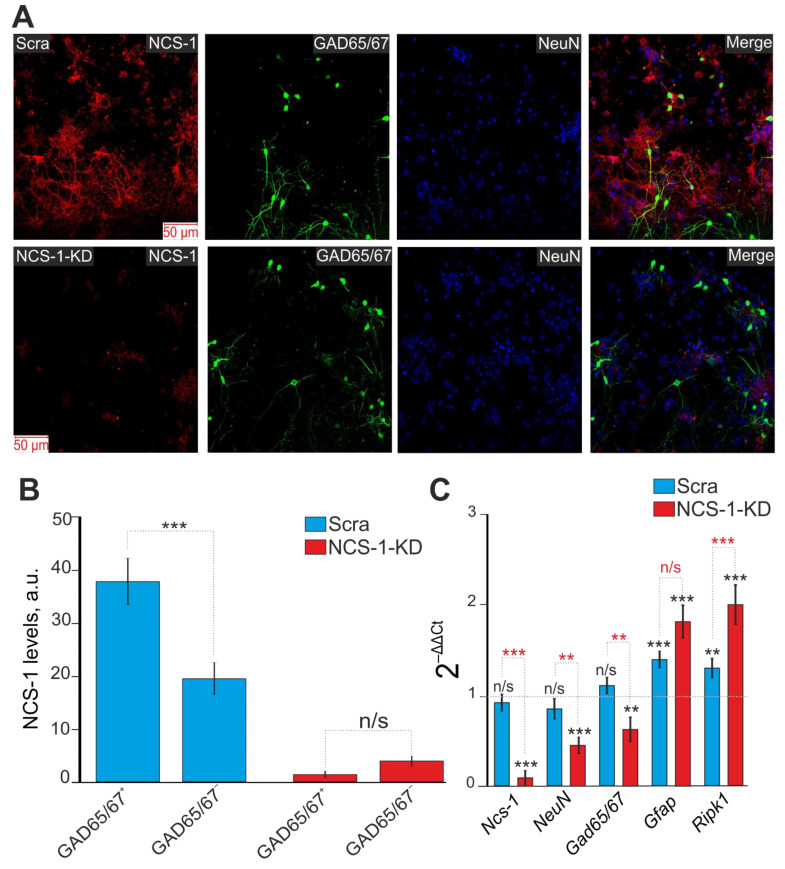
Effect of NCS-1 knockdown on the level of NCS-1 in GABAergic neurons and expression of genes encoding proteins-markers of neurons (*NeuN*), astrocytes (*Gfap*) and the pathway of necrotic death. (**A**) Immunocytochemical staining of a cell culture of the mouse cerebral cortex with antibodies against NCS-1, GAD65/67 and NeuN in control (Scra) and in cells with NCS-1 knockdown (NCS-1). Merge—the overlay of antibody fluorescence. (**B**) Level of NCS-1 expression in GABAergic (GAD65/67^+^) and non-GABAergic (GAD65/67^–^) neurons of the cerebral cortex in control samples (Scra) and in cells with NCS-1-KD. Intensity levels of NCS-1 expression were determined by confocal imaging. The quantative data reflecting the level of NCS-1 expression are presented as fluorescence intensity values in summary bar charts (mean ± SEM). The values were averaged by 150 neurons for each column. We used the scans from three independent view fields for each experimental group. Statistical significance was assessed using paired t-test. (**C**) Level of gene expression in Scra and NCS-1-KD samples in mouse cortex cells. Gene expression in control cells (without exposure to lipofectamine and shRNA) are marked by dashed line. Comparison of experimental groups regarding control: n/s—data not significant (*p* > 0.05), ** *p* < 0.01 and *** *p* < 0.001. Comparison of experimental groups relative to each other is indicated in red. The number of RNA samples is 3. N (number of animals used for cell cultures preparation) = 3.

**Figure 3:**
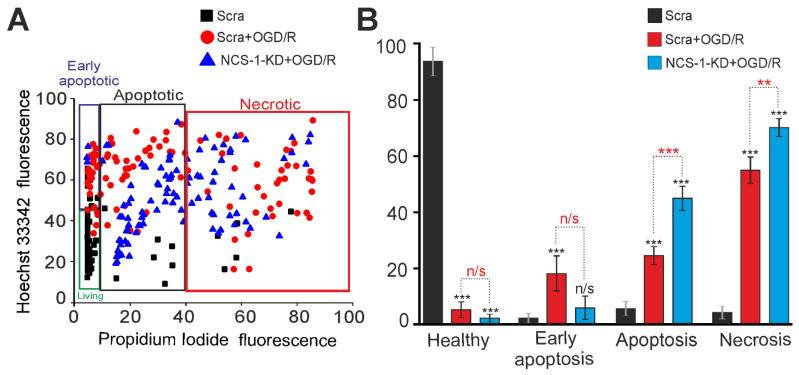
Effect of NCS-1-KD on the viability of cortical cells after 2 h of OGD exposure and 24 h of reoxygenation (OGD/R conditions). (**A**) Cytogram demonstrating the viability of cortical cells in the Control (Scra, without OGD/R) and after OGD/R in the Scra and NCS-1-KD groups. (**B**) Effects of NCS-1-KD on the induction of necrosis and apoptosis after 24 h OGD/R. Viable cells are not permeable to PI, while Hoechst 33,342 penetrates through the plasma membrane, staining the chromatin. Cortical cells were defined as apoptotic if the intensity of Hoechst 33,342 fluorescence was 3–4 times higher compared to Hoechst 33,342 fluorescence in healthy cells, indicating chromatin condensation, which can occur as a result of apoptosis induction. The differences between the early and late stages of apoptosis were determined by the intensity of Hoechst 33,342 fluorescence, and at the later stages of apoptosis, cells begin to show insignificant membrane permeability for PI. Statistical significance was assessed using unpaired t-test. Comparison of experimental groups relative to Scra: n/s—data not significant (*p* > 0.05) and *** *p* < 0.001 marked in black. Comparison of Scra + OGD/R with NCS-1-KD + OGD/R: n/s—data not significant (*p* > 0.05), *** *p* < 0.001 and ** *p* < 0.01, marked in red. The number of samples is 5. N (number of animals used for cell cultures preparation) = 3.

Cell cultures transfected with the siRNA, which sequence differed from the sequence of siRNA against NCS-1 (Figure 2A,B—Scra), did not significantly affect the expression level of NCS-1. Real-Time PCR showed that the level of expression of the gene encoding *Ncs-1* in the experimental group did not differ significantly from control cells (Figure 2C). In addition, in the Scra group, there was no significant change in the genes encoding *Ncs-1*, *NeuN*, and *Gad65/67* with an increase in the expression level of *Gfap*, a marker of astrocytes (Figure 2C). However, there was an increase in *Ripk1* gene expression by 37%, which is a sign of some activation of necrotic processes in the cell culture, which is probably associated with manipulations with the use of lipofectamine RNAiMAX (Figure 2C). NCS-1-KD in cells led to a decrease in the amount and protein of NCS-1 in both GAD65/67^−^ and GABAergic neurons of the cerebral cortex (Figure 2A–C), as well as to a two-fold increase in the level of *Ripk1* expression, compared with control, which indicates increased necrosis in the experimental group (Figure 3C).

Thus, NCS-1 is expressed predominantly in neurons of the cerebral cortex and to a much lesser extent in astrocytes. Moreover, for GABAergic neurons, a much higher level of NCS-1 expression was observed. Cellular knockdown of NCS-1 led to a decrease in the expression of genes encoding *NeuN* and *Gad65/67*, markers of neurons and the GABAergic type of neurons, respectively, occurring against the background of increased expression of *Ripk1*, a marker of necrosis induction. These data may indicate the activation of basic cell death of neurons upon NCS-1 knockdown without damage to astrocytes, since the level of *Gfap* gene expression, an astrocytic marker, even increased in the absence of NCS-1. In addition, the expression of NCS-1 in astrocytes is weakly expressed and the knockdown of this protein probably does not affect the survival of astrocytes at rest (without additional external stimulation).

### 2.2. Expression of NCS-1 Protects Neurons from OGD/Reoxygenation (OGD/R)-Induced Death through the Regulation of Apoptotic and Inflammatory Protein Expression

To study the neuroprotective role of NCS-1, a 2-h oxygen-glucose deprivation (OGD) model followed by 24-h reoxygenation (R) was used. After exposure to OGD/R, cells were stained simultaneously with Hoechst 33,342 and Propidium iodide fluorescent probes, which made it possible to establish the induction of various stages of apoptosis and necrosis. Cell cultures transfected with the Scra-siRNA did not cause massive cell death in the cerebral cortex, while 3% and 8% of cells were recorded at the early and late stages of apoptosis, and necrosis was detected in 6% of cells, respectively (Figure 3A,B—Scra). Further, 24 h after OGD/R, in the Scra experimental group, the activation of the early and late stages of apoptosis occurred in 20% and 24% of cells, respectively, and necrosis-type death was recorded in 57% of cells (Figure 3A,B—Scra + OGD/R). The knockdown of NCS-1 increased the number of cells at the late stage of apoptosis after OGD/R up to 48% with a trend towards a decrease in the percentage of cells at the early stage of apoptosis (8%). At the same time, necrosis also increased and 78% of cells with this type of death were recorded (Figure 3A,B—NCS-1-KD + OGD/R).

**Figure 4:**
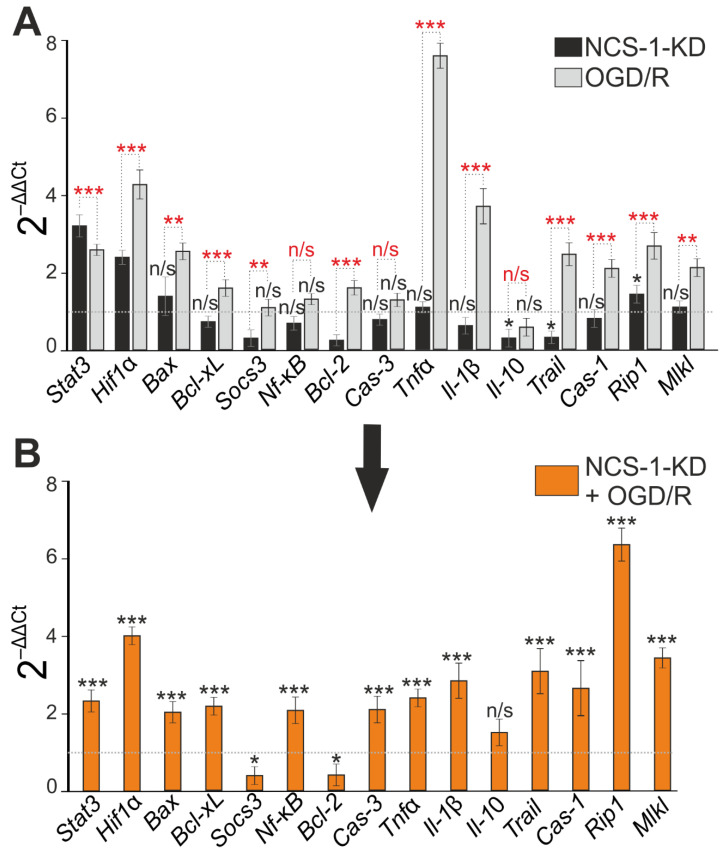
Effect of NCS-1-KD and OGD/R on mRNA expression of proteins regulating apoptosis, necrosis, and inflammation. (**A**) Baseline mRNA expression in NCS-1-KD cortical cells (black bars) and cortical cells (Scra) 24 h after OGD/R. Dashed line level of gene expression in control (Scra without NCS-1-KD and OGD/R). (**B**) Effect of NCS-1-KD on OGD/R induced gene expression. Dashed line level of gene expression in OGD/R experimental group (Scra without NCS-1-KD). Statistical significance was assessed using unpaired *t*-test. n/s—data not significant (*p* > 0.05), * *p* < 0.05, ** *p* < 0.01, and *** *p* < 0.001. Comparison between experimental group NCS-1-KD and experimental group OGD/R is marked by red asterisks. In panel (**A**), columns without black asterisks—differences are significant *** *p* < 0.001. The number of samples is 4. N (number of animals used for cell cultures preparation) = 4.

NCS-1-KD did not lead to a significant increase in the expression of pro-apoptotic genes, but caused suppression of anti-apoptotic genes Socs3 and Bcl-2 expression (Figure 4A). In addition, during NCS-1-KD, there was a decrease in the expression of the anti-inflammatory cytokine *Il-10* and, at the same time, an increase in the expression levels of the *Rip1*, *Stat3*, and *Hif1α* genes (Figure 4A, black bars). Similarly, 24 h after ischemia/reoxygenation (OGD/R), there was a trend towards an increase in the expression of *Stat3* and *Hif1α* (Figure 4A, light gray bars), but with OGD/R, an increase in the expression of pro-apoptotic genes *Bax*, *Bcl-xL*, inflammatory *Tnfα* was observed. *Il-1β* and genes encoding necrosis activating proteins *Trail*, *Cas-1*, *Rip1*, *Mlkl* (Figure 4A, light gray bars). However, OGD/R triggered a cascade of changes in genome expression aimed at induction of cell death—an increase in the expression of pro-apoptotic genes *Bax*, *Bcl-xL*, inflammatory *Tnfα*, *Il-1β* and genes encoding necrosis activating proteins *Trail*, *Cas-1*, *Rip1*, *Mlkl* (Figure 4A, light gray bars).

Modeling of OGD/R in cortical cells with NCS-1 knockdown led, in general, to an increase in the expression of all studied genes encoding cell death and inflammation proteins, compared with cells without NCS-1 knockdown exposed to ischemia/reoxygenation (Figure 4B). At the same time, expression of the protective genes *Socs3* and *Bcl-2* was suppressed (Figure 4C). However, it should be noted that the trend towards maintaining a high level of expression of the protective *Stat3* and *Hif1α* genes persisted after NCS-1 knockdown and after exposure to OGD/R.

Thus, NCS-1-KD in neurons of the cerebral cortex reduced the basal expression of genes encoding protective proteins, but significantly increased the expression of only one gene encoding RIP1 involved in cell death. At the same time, no significant increase in cell death was recorded. However, exposure of cells with NCS-1-KD to 2-h oxygen-glucose deprivation and 24-h reoxygenation led to an increase in the expression of all the studied genes encoding cell death activator proteins and the suppression of the expression of protective genes. Such a change in gene expression led to a significant increase in cell death, primarily due to an increase in the percentage of cells at the late stage of apoptosis and induction of necrosis.

### 2.3. Expression of NCS-1 Protects Neurons from OGD/R-Induced Death through the Regulation of Calcium-Binding Protein Expression and Cytosolic Calcium Concentration

**Figure 5:**
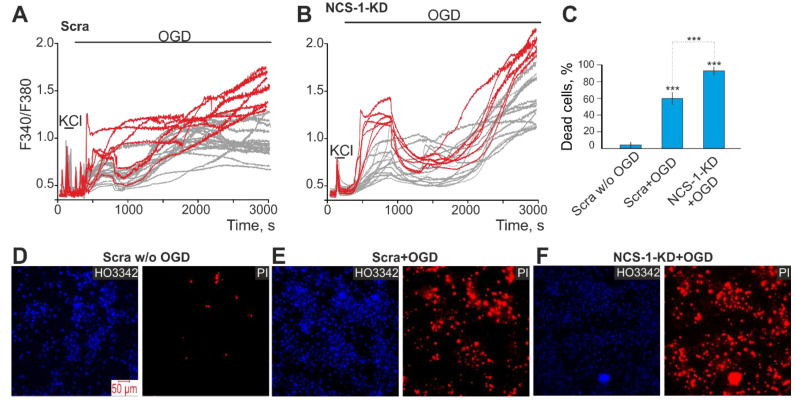
Effect of NCS-1-KD on OGD-induced Ca^2+^-signals of non-GABAergic (GAD65/67^–^) and GABAergic neurons of the cerebral cortex and their death. (**A**, **B**) Ca^2+^-signals of GABAergic (red curves) and GAD65/67^–^ (gray curves) neurons during a 40-min OGD in control (Scra, (**A**)) and after NCS-1-KD (NCS-1-KD, (**B**)). (**C**) The average number of PI-stained cortical cells that died due to OGD-induced necrosis in the Scra-group and NCS-1-KD-group. Statistical significance was assessed using paired t-test. Comparison Scra + OGD and NCS-1-KD + OGD with Scra w/o OGD significant, *** *p*-level < 0.001. (**D**–**F**) Images of cortical cell culture in the Hoechst 33,342 (HO342) and Propidium Iodide (PI) fluorescence detection channel in the experimental group without OGD ((**D**) Scra w/o OGD) and after 40-min OGD treatment in Scra group ((**E**) Scra + OGD) and neuronal calcium sensor-1 knockdown group ((**F**) NCS-1-KD + OGD). The red dots represent the PI-stained nuclei of necrotic cells. The number of samples is 6. N (number of animals used for cell cultures preparation) = 3.

We have previously shown that neuronal death in acute OGD occurs due to a global increase in [Ca^2+^]_i_ [23], while GABAergic neurons are the most sensitive to OGD and die first [2]. Baseline cell death by necrosis type in the control group (Scra) without OGD averaged. The induction of OGD for 40 min in control cells caused a biphasic increase in [Ca^2+^]_i_ in cortical neurons. The first phase of the increase in [Ca^2+^]_i_ was fully or partially reversible, while the second phase was a global increase in [Ca^2+^]_i_ (Figure 5A,B). After registration of [Ca^2+^]_i_ during OGD, the cells were fixed and stained with antibodies against GAD65/67 and compared with [Ca^2+^]_i_ signals according to the scheme shown in Figure 10. GABAergic neurons (positively stained for GAD65/67) are characterized by a greater increase in [Ca^2+^]_i_ during both phases of OGD (Figure 5A, red curves) compared to GAD65/67^−^ neurons (Figure 5A, gray curves). After 40 min of OGD, 59 ± 12% of cells died in the Scra group (Figure 5C,E—Scra + OGD). NCS-1-KD led to the induction of a more pronounced increase in [Ca^2+^]_i_ during the first and second phases of Ca^2+^-signals under OGD conditions. Moreover, in GABAergic neurons with NCS-1-KD, the first phase of the [Ca^2+^]_i_ increase is especially pronounced (Figure 5C, red curves) compared to neurons in the Scra experimental group. In cells with NCS-1 knockdown, the number of dying cells increased after OGD due to necrosis up to 83 ± 8% (Figure 5C,F).

**Figure 6:**
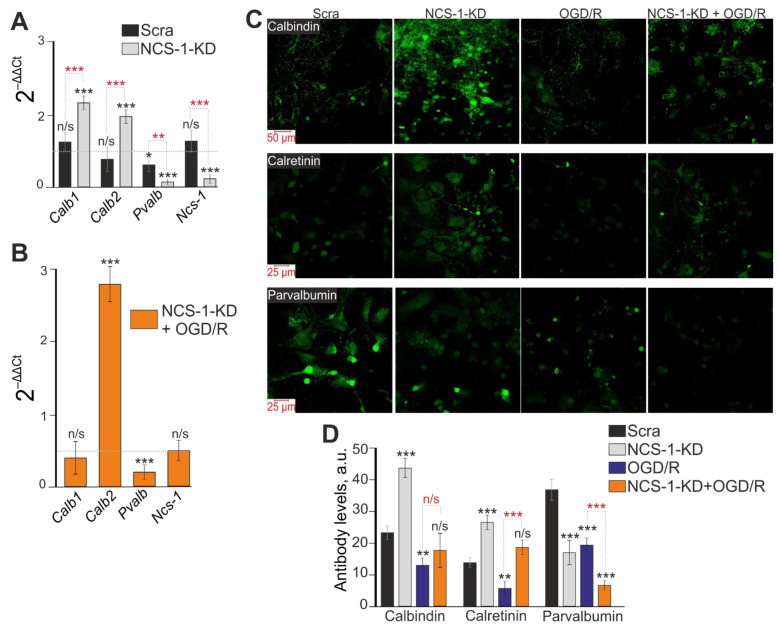
Effect of NCS-1-KD on basal and OGD/R-induced expression of calcium-binding proteins calbindin (Calb1), calretinin (Calb2), and parvalbumin (Pvalb). (**A**) Basic expression of genes encoding calcium-binding proteins in the Scra experimental group (siRNA, which sequence differed from the sequence of siRNA against NCS-1) and NCS-1-KDcells (NCS-1-KD). Dashed line level of gene expression in control. Statistical significance was assessed using paired *t*-test compared to Control cells (marked with black asterisks). Comparison between experimental group Scra and experimental group NCS-1KD is marked by red asterisks. n/s—data not significant (*p* > 0.05), * *p* < 0.05, ** *p* < 0.01, and *** *p* < 0.001. (**B**) Effect of NCS-1-KD on OGD/R-induced expression of genes encoding calcium-binding proteins. Dashed line level of gene expression in OGD/R group (Scra without NCS-1 knockdown). Statistical significance was assessed using paired *t*-test. n/s—data not significant (*p* > 0.05) and *** *p* < 0.001. (**C**) Immunostaining cortical cells with antibodies against Calbindin, Calretinin and Parvalbumin in control (Scra), NCS-1-KDgroup (NCS-1-KD), 24 h after OGD/R in control group (OGD/R) and cells with NCS-1-KD (NCS-1-KD + OGD/R). (**D**) Intensity levels of antibodies were determined by confocal imaging. We analyzed individual cells which had fluorescence of secondary antibodies. The quantitative data reflecting the level of Calbindin, Calretinin and Parvalbumin expression are presented as fluorescence intensity values in summary bar charts (mean ± SEM). The values were averaged by 100 cells for each column. The results obtained after immunostaining agree well with the data of fluorescent presented in panels (**C**). Statistical significance was assessed using paired *t*-test compared to Scra (marked with black asterisks). Comparison between experimental group OGD/R and experimental group NCS-1KD+OGD/R is marked by red asterisks. n/s—data not significant (*p* > 0.05), ** *p* < 0.01, and *** *p* < 0.001. For repeats, 4 separate cell cultures were used. N (number of animals used for cell cultures preparation) = 4.

We have previously shown that calcium-binding proteins calbindin, calretinin, and parvalbumin protect GABAergic neurons from death during hypoxia/ischemia [24]. Using PCR analysis, it was possible to establish that NCS-1-KD led to an increase in the expression of genes encoding calbindin (*Calb1*) and calretinin (*Calb2*), but significantly suppressed the expression of parvalbumin (*Pvalb*) (Figure 6A, NCS-1-KD). At the same time, the use of siRNA, which sequence differed from the sequence of siRNA against NCS-1 (Scra), significantly reduced the expression of the gene encoding parvalbumin (Figure 6A, Scra), but did not affect other genes. There was an increase in the expression of the gene encoding calretinin (*Calb2*) and a decrease in the expression of parvalbumin (*Pvalb*) in the experimental group with NCS-1-KD after 24 h OGD/R, compared with control cells (Figure 6B). The results of PCR analysis are in good agreement with the data of immunocytochemical staining (Figure 6C,D). It can be seen that the level of calbindin protein increased in NCS-1-KD cells (Figure 6C—top, Figure 6D) and significantly decreased after OGD/R in control cells, but did not change in the NCS-1-KD group after OGD/R (Figure 6C,D). It was similarly observed for calretinin when its level was higher in the NCS-1-KD group, but did not change in the same cells after OGD/R compared to the control (Figure 6C—middle line, Figure 6D). Whereas OGD/R in control cells led to a decrease in the content of calretinin (Figure 6D). Intracellular parvalbumin levels decreased in all experimental groups, but a particularly pronounced effect was observed in NCS-1-KD cortical cells 24 h after OGD/R (Figure 6C—bottom line, Figure 6D).

Thus, NCS-1-KD in cerebral cortex cells led to an increase in the number of cerebral cortex neurons dying during ischemia (OGD) due to the death of the most sensitive population of GABAergic neurons, since a more massive increase in [Ca^2+^]_i_ was observed in them. Damage primarily to GABAergic neurons during ischemia/reoxygenation correlates well with suppression of the expression of the key protective calcium-binding protein, parvalbumin, even with an increase in the expression of mRNA of two other proteins, calbindin and calretinin.

### 2.4. The Mechanism of the Neuroprotective Action of NCS-1 Occurs Due to the Suppression of Hyperexcitation of GABAergic Neurons and the Suppression of the Global [Ca^2+^]_i_ Increase

**Figure 7:**
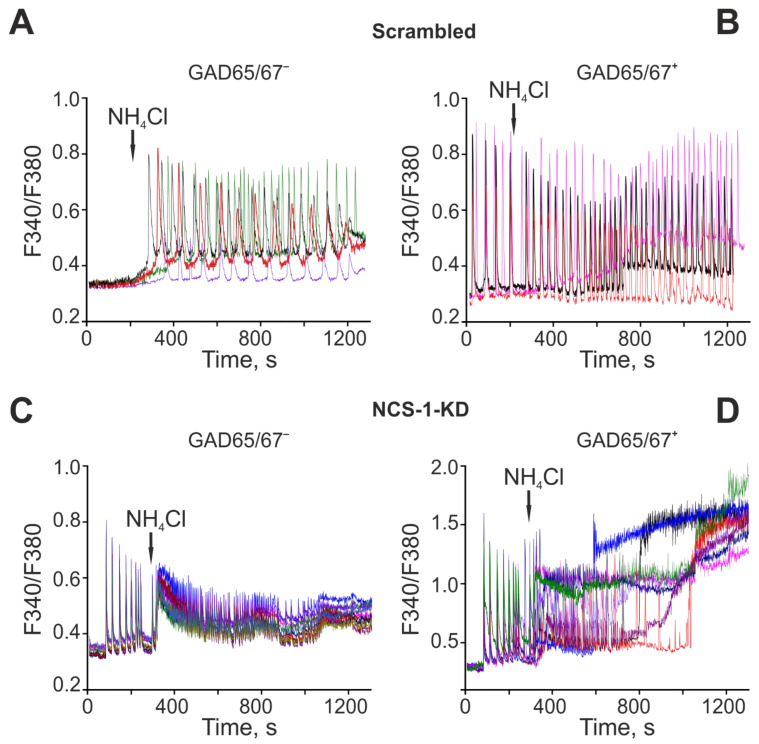
The role of NCS-1 in hyperexcitation of non-GABAergic (GAD65/67^–^) and GABAergic neurons of the cerebral cortex under the action of hyperammonemia. NCS-1 contributions to NH_4_Cl-induced global [Ca^2+^]_i_ increase in GABAergic neurons. (**A**, **B**) Ca^2+^-signals of control (Scra) non-GABAergic (GAD65/67^–^) (**A**) and GAMBergic (**B**) neurons of the cerebral cortex upon application of 8 mM NH_4_Cl. (**C**, **D**) Ca^2+^-signals of non-GABAergic (**C**) and GABAergic (**D**) neurons of the cerebral cortex after NCS-1-KD upon application of 8 mM NH_4_Cl. Typical Ca^2+^-signals of neurons in one experiment are shown.

Since NCS-1-KD had a pronounced effect on the generation of the first phase of the OGD-induced increase in [Ca^2+^]_i_ in GABAergic neurons, which is a sign of hyperexcitation of the network, it was decided to test the role of NCS-1 in hyperexcitation of the neuronal network in a model of acute hyperammonemia. In control (Scra) non-GABAergic (GAD65/67^–^) neurons of the cerebral cortex, the application of 8 mM NH4Cl caused the generation of non-synchronous Ca^2+^ oscillations that occurred with an increase in the base concentration of [Ca^2+^]_i_ (Figure 7A). The population of GABAergic neurons (GAD65/67^+^) is characterized by the presence of spontaneous synchronous Ca^2+^-oscillations in culture. At the same time, an application of 8 mM NH_4_Cl led to an increase in the frequency of oscillations and, in individual neurons, to a gradual increase in the base concentration of [Ca^2+^]_i_ (Figure 7C). NCS-1-KD cardinally changed the behavior of the Ca^2+^ signaling system of non-GABAergic and GABAergic neurons in the cerebral cortex. In both types of neurons, spontaneous synchronous Ca^2+^-oscillations were observed, the amplitude of which was higher in GABAergic neurons (Figure 7D). An application of NH_4_Cl to non-GABAergic (GAD65/67^–^) neurons resulted in a synchronous increase in the base concentration of [Ca^2+^]_i_ and the subsequent inhibition of Ca^2+^-oscillations (Figure 7C). In GABAergic neurons, the application of NH_4_Cl contributed to a rapid desynchronization of the network and, most importantly, to a global [Ca^2+^]_i_ increase leading to death (Figure 7D).

**Figure 8:**
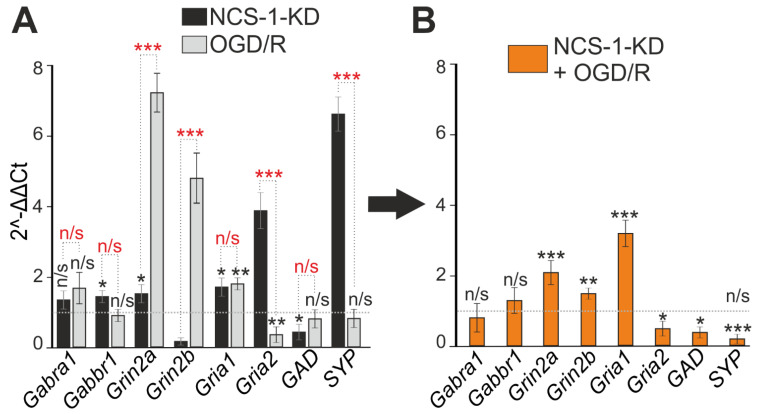
Effect of NCS-1-KD and OGD/R knockdown on the expression of genes encoding proteins-regulators of neuronal hyperexcitation. (**A**) Baseline gene expression in NCS-1-KD cortical cells (black bars) and control (Scra) cells 24 h after OGD/R (gray bars). Dashed line level of gene expression in control (Scra without NCS-1-KD and OGD/R). (**B**) Effect of NCS-1-KD on OGD/R induced gene expression. Dashed line level of gene expression in OGD/R experimental group (Scra without NCS-1-KD). Statistical significance was assessed using unpaired t-test. n/s—data not significant (*p* > 0.05), * *p* < 0.05, ** *p* < 0.01, and *** *p* < 0.001. In panel (**A**), columns without asterisks—differences are significant *** *p* < 0.001. Comparison between experimental group NCS-1-KD and experimental group OGD/R is marked by red asterisks. The number of samples is 4. N (number of animals used for cell cultures preparation) = 4.

The formation of symptoms of hyperexcitation of the neuronal network in both hyperammonemia and ischemia is largely responsible for the balance of expression of excitatory and inhibitory receptors. It turned out that NCS-1-KD did not affect the expression of genes (*Gabra1* and *Gabbr1*) encoding subunits of inhibitory GABA receptors, but led to the suppression of the expression of *Gad* encoding glutamate decarboxylase 65/67, which may contribute to a decrease in the synthesis of the inhibitory mediator γ-aminobutyric acid (Figure 8A). In addition, NCS-1-KD correlated with an increase in the expression of the *Gria1* and *Gria2* genes encoding AMPA receptors and the *Grin2a* gene encoding the NMDA receptor subunit (Figure 8A). The effect of NCS-1-KD on the expression of genes encoding inhibitory regulatory proteins in the neuronal network also persisted 24 h after OGD/R. At the same time, the expression of GABA receptors did not change, but the level of glutamate decarboxylase 65/67 decreased. In addition, an increase in the expression of genes encoding subunits of NMDA receptors, the *Gria1* subunit of AMPA receptors was observed, which occurred against the background of suppression of the expression of the Ca^2+^-conducting *Gria2* subunit of AMPA receptors (Figure 8C). Interestingly, the expression level of the *Syp* gene encoding synaptophysin, a key nerve ending protein, increased upon NCS-1-KD (Figure 8A), but decreased after OGD/R (Figure 8B) compared with control cells, which may indicate damage to neurotransmission during ischemia/oxygenation.

Thus, NCS-1-KD contributed to the formation of hyperexcitation of the neuronal network during ischemia and hyperammonemia, leading to damage primarily to GABAergic neurons due to the switching of high-amplitude Ca^2+^-oscillations into a global irreversible increase in [Ca^2+^]_i_. The mechanism for the formation of this hyperexcitation involves an increase in the expression of excitatory ionotropic glutamate receptors NMDA and AMPA, the suppression of the expression of glutamate decarboxylase 65/67 and the synthesis of γ-aminobutyric acid.

**Figure 9:**
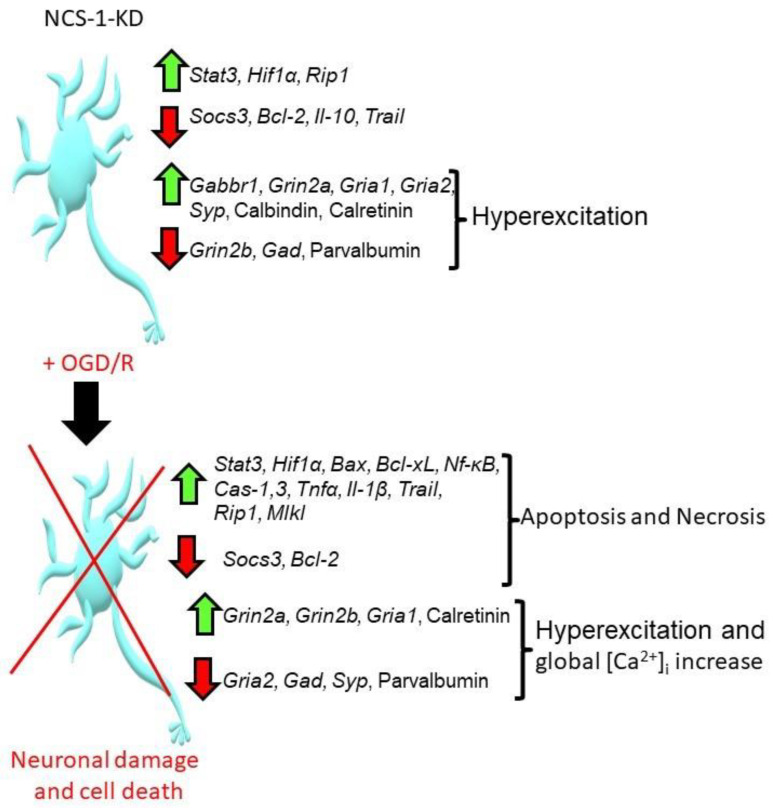
Summarizing scheme of the effects of NCS-1 knockdown in the cortical cells under the action of OGD/R. Abbreviations: *Stat3*—signal transducer and activator of transcription 3, *Hif1α*—Hypoxia-inducible factor 1-alpha, *Rip1*—Receptor interacting protein kinase 1, *Socs3*—Suppressor of cytokine signaling 3, *Bcl-2*—B-cell lymphoma 2, *Il-10*—Interleukin-10, *Trail*—TNF-related apoptosis-inducing ligand, *Tnfα* — Tumor necrosis factor alpha, *Gabbr1*—Gamma-aminobutyric acid (GABA) B receptor, 1, *Grin2a*—Glutamate Ionotropic Receptor NMDA Type Subunit 2A, *Grin2b*—Glutamate Ionotropic Receptor NMDA Type Subunit 2B, *Gria1*—Glutamate Ionotropic Receptor AMPA Type Subunit 1, *Gria2*—Glutamate Ionotropic Receptor AMPA Type Subunit 2, *Syp*—Synaptophysin, *Gad*—Glutamic acid decarboxylase, *Bax*—bcl-2-like protein 4, *Bcl-xL*—B-cell lymphoma-extra-large, *Cas-1, 3*—caspase 1 and 3, *Il-1β*—Interleukin-1β, *Mlkl*—Mixed Lineage Kinase Domain Like Pseudokinase, *Nf-κB*—nuclear factor kappa-light-chain-enhancer of activated B cells.

Figure 9 summarizes the effect of NCS-1 knockdown on cortical neurons under ischemia-like conditions and reoxygenation (OGD/R). The knockdown of NCS-1 led to changes in gene expression, leading predominantly to the hyperexcitation of neurons and especially GABAergic neurons. At the same time, such neurons mainly survived in culture when they were not exposed to toxic conditions. However, neurons with NCS-1 knockdown were characterized by increased sensitivity to damage during OGD/R, when there was an increased expression of pro-apoptotic genes, a decreased expression of protective genes, and a calcium-binding protein, parvalbumin. The action of OGD, OGD/R or hyperammonemia led to hyperexcitation of neurons leading to a global increase in [Ca^2+^]_i_, the activation of apoptosis and necrosis.

## 3. Discussion

Deficiency of NCS-1 in the brain is not lethal in mice and does not cause obvious abnormalities during embryonic development. However, NCS-1 KO mice display depressive-like behavior, as these mice spent more time immobile in forced swim and tail suspension behavioral tests [25]. Whereas in mice, the deletion of the NCS-1 gene resulted in 30% mortality in newborns during the first 4 days of life [26]. Accordingly, the expression of NCS-1 not only affects the behavior and emotional state, but also the survival of the organism. Hyperexcitation or depressive-like behavior at the cellular level is determined by the balance between excitation and inhibition in the neuronal networks of the brain. The excitatory component of neurotransmission is determined by glutamatergic neurons, the key neurotransmitter of which is glutamate, and the inhibitory component is determined by GABAergic neurons secreting γ-aminobutyric acid. As shown by our experiments and the works of other authors, NCS-1 is expressed both in glutamatergic and GABAergic neurons and even to a small extent in glial cells [27,28]. It has been shown that neurons expressing calcium-binding proteins (CaBPs)—calbindin (CB) and parvalbumin (PV) contain large amounts of NCS-1, while neurons without these proteins express NCS-1 at a significantly lower level. In view of the high homology between calbindin and calretinin (CR), a similar trend can be assumed for calretinin. However, synaptophysin-positive boutons typically do not contain NCS-1. At the same time, GABAergic neurons are characterized by an increased content of calbindin, parvalbumin, and NCS-1 [27]. It is also known that NCS-1 potentiates the release of neurotransmitters from excitatory synapses in a cell culture of hippocampal neurons [16], and the presence of NCS-1 in GABAergic neurons may also suggest the participation of this protein in the modulation of inhibition through the release of GABA [27].

The neuroprotective role of calbindin, calretinin, and parvalbumin has been shown for GABAergic neurons. These proteins determine the lower sensitivity of GABAergic neurons to glutamate and contribute to the survival of these neurons during episodes of reoxygenation due to the significant resistance to posthypoxic hyperexcitation of the network. Additionally, during ischemia, these CaBPs have a protective effect on GABAergic neurons, inhibiting the appearance of the second phase of the response to OGD and regulating the rate of increase in [Ca^2+^]_i_ in this phase [24,29]. CR and CB (Calbindin-D28k) are fast CaBPs capable of binding Ca^2+^ immediately after its concentration in the cytosol increases. They are 58% identical in structure. Ca^2+^-binding affinity for calbindin (Kd ∼0.5–1.5 µM) and for calretinin (Kd ∼0.5–1.2 µM) [30]. Parvalbumin, unlike the other two proteins, has a slow binding kinetics of Ca^2+^ ions and Kd for Ca^2+^ ions is ~0.01–0.1 µM [31]. At the same time, NCS-1 displays a high Ca^2+^-binding affinity (Kd for Ca^2+^ ~200–300 nM) and is able to respond to any fluctuations in [Ca^2+^]_i_ above resting levels [32], which also suggests its involvement in the protection of neurons from damage under various influences.

It is known that excessive entry of Ca^2+^ ions into the cytosol under stress conditions leads to cell damage and death. Whereas a moderate increase in [Ca^2+^]_i_ through sensor proteins activates protective signaling cascades. It has been established that a low level of NCS-1 expression in neurons of the pedunculopontine nucleus is accompanied by gamma oscillations, and overexpression of NCS-1 leads to their suppression. In addition, the application of exogenous NCS-1 reduces the amplitude of these oscillations in neurons [33]. Additionally, in our experiments, the knockdown of NCS-1 leads to the appearance of spontaneous Ca^2+^-oscillations in the non-GABAergic and GABAergic neurons of the cerebral cortex, which was not observed in the control. The cytoprotective mechanisms of NCS-1 have not been sufficiently studied so far. However, NCS-1 has been reliably shown to regulate neurite outgrowth in pond snails [13] and in primary cultured embryonic chick dorsal root ganglia neurons [34]. Neuronal studies have shown that the overexpression of NCS1 induces neurite sprouting associated with increased phospho-Akt levels. When the PI3K/Akt signaling pathway was pharmacologically inhibited, the NCS1-induced neurite sprouting was abolished [15]. At the same time, the signaling cascade involving PI3K is a key in the survival of GABAergic neurons during hypoxia or ischemia [35,36]. In addition, we have shown that in cortical cells with NCS-1 knockdown after ischemia/reoxygenation, there is a significant decrease in the expression of synaptophysin, which forms synapses. Our results obtained on neurons of the cerebral cortex when modeling ischemia/reoxygenation are in good agreement with the results obtained on cardiomyocytes. It has been shown that cardiomyocytes derived from NCS-1^−/−^-mice are more sensitive to metabolic and oxidative stress, since they are characterized by a reduced level of ATP and impaired mitochondrial respiration. Such cardiomyocytes died under ischemia/reperfusion conditions, and Akt and PGC-1α activators contributed to their survival to some extent [37].

NCS-1 was found to be a Ca^2+^ -dependent survival-promoting factor in injured neurons, since its expression is increased in injured neurons and the overexpression of NCS-1 protects cells from death, while the dominant negative EF-hand NCS-1 mutant (E120Q) accelerated cell death. It has been shown that the level of NCS-1 expression regulates the level of GDNF in neurons through a signaling cascade involving Akt [38]. In cardiac tissue, NCS-1 protects cardiomyocytes from damage through the activation of mitochondrial detoxification signaling pathways [37]. Ncs-1^−/−^-myocytes die as a result of oxidative and metabolic stress due to the depletion of ATP reserves, a decrease in mitochondrial respiration and biosynthesis, and a decrease in the expression of Akt and PGC-1α [38]. It is known that NCS-1 is associated with the regulation of adenosine receptor activity, which, in turn, is associated with the induction of ischemic preconditioning [39,40]. The overexpression of NCS-1 contributes to the formation of greater tolerance of cortical neurons to oxidative stress and nutritional deficiencies during in vitro cultivation due to the suppression of apoptosis through an increase in GDNF expression [14]. In our experiments, NCS-1-KD itself did not lead to the massive death of neurons in the cerebral cortex, but as a result of acute ischemia (OGD for 40 min) or 24 h after OGD/R, a significant increase in apoptosis and necrosis was observed. There are only sporadic studies on the role of NCS-1 in the response of nervous system cells to hypoxia/ischemia. NCS-1 has been shown to be upregulated in the central nervous system of the freshwater pond snail, *Lymnaea stagnalis*, following chronic hypoxia treatment. This upregulation coincides with increased aerial respiratory activity. Furthermore, NCS-1-KD attenuates hypoxia-induced facilitation of aerial respiration. The knockdown of NCS-1 partially prevents these hypoxia-induced changes [41]. Therefore, NCS-1 expression is required for the adaptation of the nervous system to hypoxia. NCS-1 has been shown to directly interact with the yeast orthologue of PI4K, Pik1, and this is essential for survival in yeast [42]. NCS-1 has also been shown to coimmunoprecipitate with mammalian PI4K from COS-7 cells [43], bovine chromaffin cells [44] and rat neuro-secretory cells [45]. NCS-1 is involved in the regulation of cell survival pathways not only through Akt and PI3K, but also through Phosphatidylinositol 4-kinase β, whose activity is regulated by changes in [Ca^2+^]_i_.

There is currently no data on the role of NCS-1 in the regulation of glutamate receptor activity, but it has been shown that NCS-1 appears to facilitate P/Q-type calcium channel currents [46], which are involved in the generation of Ca^2+^-signals during activation of AMPA and NMDA receptors. NCS-1 interacts with a plethora of target molecules in the central nervous system and has a half-maximal affinity for calcium lower than 1 μM [47]. NCS-1 has been shown to act as a calcium sensor in short-term plasticity in rat hippocampal cell cultures, switching paired pulse depression to paired pulse facilitation without altering basal transmission or initial transmitter release probability, probably by recruiting ‘dormant’ vesicles [16]. Long-term depression is regulated by an increase in [Ca^2+^]_i_, which occurs at the expense of ionotropic NMDA receptors and metabotropic mGluR receptors. Ncs-1^−/−^-mice exhibit behavioral amphetamine sensitization, suggesting that NCS-1 plays a role in a type of long-term depression and AMPA receptor endocytosis in the paranasal cortex [18]. Additionally, in our experiments, NCS-1-KD in the neurons of the cerebral cortex did not affect the expression of subunits that form NMDA receptors, but their expression increased during OGD/R both in control cells and in the NCS-1-KD group, which may be the effect of ischemia/reoxygenation only. At the same time, there is evidence of a close relationship between NCS-1 and the regulation of AMPA receptors. Ncs-1^−/−^-mice show behavioral sensitization to amphetamine suggests that while NCS-1 has a role in a type of long-term depression and AMPA receptor endocytosis in the perirhinal cortex [18]. In addition, NCS-1 is associated with the plasma membrane via its myristoylated and is involved in initiate AMPA receptor removal from the synapse [47]. In our experiments, NCS-1 knockdown promoted the expression of subunits that form the AMPA receptor and especially the calcium-conducting Glua2 subunit after OGD/R, which correlated with an increase in hyperexcitation, especially in GABAergic neurons.

As our studies and analysis of the work of other researchers show, we can conclude that the expression of NCS-1 in neurons is involved in the regulation of a wide range of vital processes. In the present study, we touched upon only a small part of the signaling pathways that are regulated by NCS-1. However, we clearly established the role of NCS-1 in protecting GABAergic neurons, and, consequently, other neurons of the neuronal network from hyperexcitation, ischemia-like conditions, and reoxygenation. At the same time, a serious limitation of our study is the use of exclusively in vitro models, and on the whole organism, numerous compensatory mechanisms can be implemented during NCS-1 knockout and damaging conditions.

## 4. Materials and Methods

Experimental protocols were approved by the Bioethics Committee of the Institute of Cell Biophysics. Experiments were carried out according to Act708n (23 August 2010) of the Russian Federation National Ministry of Public Health, which states the rules of laboratory practice for the care and use of laboratory animals, and the Council Directive 2010/63 EU of the European Parliament on the protection of animals used for scientific purposes.

### 4.1. Preparation of Mixed Neuroglial Cell Cultures

Cell cultures from cerebral cortex were prepared as described in detail previously [48]. Briefly, 0–1 day old pups were euthanized and decapitated. The extracted tissue was washed with Mg^2+^- and Ca^2+^-free Versene solution and minced with scissors. Then, the tissue fragments were digested with 1% trypsin solution for 10 min at 37 °C and washed two times with cold Neurobasal-A medium. Trypsinized tissue was gently triturated with a pipette, and the debris was then carefully removed with a pipette tip. The obtained cell suspension was seeded on polyethyleneimine-coated glass coverslips and grew for 10 days in vitro in the cell culture medium composed of Neurobasal-A medium, supplement B-27 (2%) and 0.5 mM glutamine.

### 4.2. Immunocytochemistry

**Figure 10:**
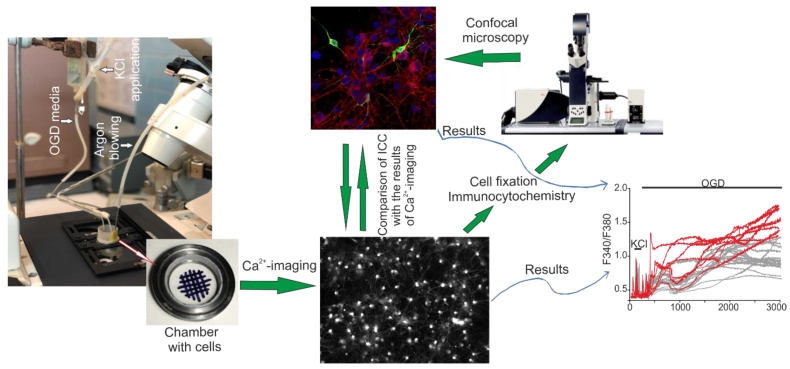
Scheme of the original setup for creating OGD conditions for cells of the cerebral cortex in vitro and a method for comparing the results of Ca^2+^ imaging with the data of immunocytochemical staining of cell cultures. Cells of the cerebral cortex were grown on round coverslips and mounted in an experimental chamber, which was mounted on the object table of an inverted fluorescence microscope. A system for supplying OGD-media and blowing inert argon gas was connected to the chamber. After recording the [Ca^2+^]_i_ dynamics, the cells were fixed and stained with specific antibodies. Next, the chamber with cells was transferred to an inverted confocal microscope, and using a grid, an area with cells was found in which the change in [Ca^2+^]_i_ was recorded. The obtained confocal images were combined with a series of images obtained using Ca^2+^ imaging. Red curves is GABAergic neurons and gray curves is non-GABA-neurons.

Coverslips with cortical cell cultures were mounted in the experimental chamber. Marker grid was plotted on the bottom side of each coverslip. The chamber was placed on the stage of the microscope, and a random grid-bordered area was chosen for the fluorescent Ca^2+^ imaging. Then, the cells were fixed and stained with antibodies according to the previously described protocol [49]. Briefly, the cells were rinsed with Ca^2+^ and Mg^2+^-free PBS and fixed for 20 min with the solution composed of 4% paraformaldehyde and 0.25% glutaraldehyde diluted in PBS. After that, the cells were rinsed thrice with ice-cold PBS and permeabilized with 0.1% Triton X-100 solution. Then, the cells were incubated for 30 min with 10% donkey serum (in PBS) to block non-specific binding of the secondary antibodies and stained overnight at 4 °C with primary antibodies diluted in 1% donkey serum. We used chicken anti-NeuN (ABN91, Sigma-Aldrich, Burlington, USA) antibodies to identify neurons, rabbit anti-GAD 65/67 antibodies (Abcam, Cambridge, UK) to discriminate GABAergic neurons, mouse NCS-1 monoclonal antibody (Cat No. 67616-1-Ig, Proteintech Group, Rosemont, IL, USA) to determine the level of neuronal calcium sensor-1 in cells and mouse monoclonal anti-GFAP antibody (RRID: AB_2632644, BioLegend, San Diego, CA, USA) to identify astrocytes. To detect calcium-binding proteins calbindin, calretinin, and parvalbumin, we used mouse monoclonal anti-Calbindin-D-28K antibody (SAB4200543, Sigma-Aldrich, Burlington, VT, USA), rabbit polyclonal Anti-Calretinin antibody (ab244299, Abcam, Cambridge, UK), rabbit polyclonal anti-Parvalbumin antibody (ab11427, Abcam, Cambridge, UK). The cells were rinsed thrice with PBS after the incubation with primary antibodies and stained with secondary antibodies. We used secondary donkey anti-rabbit Alexa Fluor-488 or Alexa Fluor-555 conjugated antibodies (1:200; Abcam, Cambridge, UK), donkey anti-mouse Alexa Fluor-647 conjugated antibodies (1:200; Abcam, Cambridge, UK) and donkey anti-chicken Alexa Fluor-488 conjugated antibodies (1:200; Abcam, Cambridge, UK). Cell nuclei were stained with Hoechst 33,342 (ab228551, Abcam, Cambridge, UK) or Draq5 (ab108410, Abcam, Cambridge, UK). Fluorescence of the conjugated dyes was detected with Leica TCS SP5 confocal microscope in the grid-bordered areas, which were chosen for Ca^2+^ imaging. The confocal images of cell cultures stained with the antibodies were matched with the images of the same cultures captured during vital Ca^2+^ imaging, as describe early [1]. Thus, the combination of vital Ca^2+^ imaging and immunostaining allows obtaining data about Ca^2+^ dynamics in NCS-1-, NeuN- and GAD65/67-positive cells (Figure 10).

### 4.3. Transfection with Small Interfering RNA (siRNA)

When cell confluence reached at 40% (5 days in vitro), cells were transfected with siRNA against mouse neuronal calcium sensor-1 (NCS-1) (abx925540, Abbexa, Cambridge, UK) using lipofectamine RNAiMax (Thermo Fisher Scientific, Waltham, MA, USA) according to the manufacturer’s instructions. After incubating cortical cells with siRNA-reagent mixtures in Opti-MEM (Gibco, Thermo Fisher Scientific, Waltham, MA, USA) containing 50 pM of siNCS-1 were added for 6 h. Then cultural medium changed and cells were incubated for an additional 48 h. The efficiency of knockdown was at least 85–90% as confirmed by RT-PCR and immunostaining with anti-NCS-1 antibodies (Figure 2).

### 4.4. Fluorescent Ca^2+^ Measurements

To detect the changes in [Ca^2+^]_i_, cell cultures were loaded with Fura-2 (4 µM; 40 min incubation; 37 °C). The cells were stained with the probe dissolved in Hank’s balanced salt solution (HBSS) composed of (mM): 156 NaCl, 3 KCl, 2 MgSO4, 1.25 KH_2_PO_4_, 2 CaCl_2_, 10 glucose, and 10 HEPES, pH 7.4. To measure [Ca^2+^]_i_, we used the system based on an inverted motorized microscope Leica DMI6000B with a high-speed monochrome CCD-camera HAMAMATSU C9100. For excitation and registration of Fura-2 fluorescence, we used FU-2 filter set (Leica, Wetzlar, Germany) with excitation filters BP340/30 and BP387/15, beam splitter FT-410, and emission filter BP510/84. Illuminator Leica EL6000 with a high-pressure mercury lamp was used as a source of excitation light. To distinguish neurons and astrocytes, we used short-term applications of 35 mM KCl before the main experiments. Briefly, KCl induces depolarization of excitable cells, which contain a wide range of voltage-gated cation channels. KCl-induced depolarization promotes the opening of voltage-gated calcium channels in neurons (predominantly L-type channels). The conductivity and density of cation channels in astrocytes are insufficient to evoke high-amplitude Ca^2+^ response to KCl application. All the Ca^2+^ signals are presented as 340/380 ratio of Fura-2 fluorescence.

### 4.5. The Technique for Simulation of Ischemia-like Conditions

Ischemia-like conditions (oxygen-glucose deprivation, OGD) were obtained by omitting glucose (HBSS medium without glucose) and by displacement of dissolved oxygen with argon in the leak-proof system [36]. The level of oxygen in the medium was measured using a Clark electrode. Oxygen tensions reached values 30–40 mm Hg or less within 20 min after the beginning of displacement. Ischemia-like conditions lasting for 40 min or 2 h were created using supplying the oxygen-glucose deprivation (OGD)-medium into the chamber with cultured cortical cells. Constant argon feed into the experimental chamber was used to prevent the contact of the OGD-medium with the atmospheric air.

### 4.6. Assessment of Cell Viability and Apoptosis

Propidium iodide (1 µM) were used to evaluate the number of dead cells in the cell cultures before and after OGD. The cells were stained for 5 min with the probes diluted in HBSS and then rinsed with HBSS. Fluorescence of the probes was detected with an inverted fluorescent microscope Zeiss Axio Observer Z1 using Filter Set 20. Cell death induced by OGD was assessed by propidium iodide staining (PI, 1 µM) before and after the exposures in the same microscopic field. Furthermore, we used the Ca^2+^ signals (presence or absence of a global increase in [Ca^2+^]_i_ during OGD) as an additional indicator of cell viability [50,51].

Hoechst 33,342 (2 µM) and propidium iodide (1 µM) were used to evaluate the number of dead cells in the cell cultures before and after 2 h OGD and 24 h reoxygenation (OGD/R conditions). The cells were stained for 5 min with the probes diluted in HBSS and then rinsed with HBSS. Fluorescence of the probes was detected with an inverted fluorescent microscope Zeiss Axio Observer Z1 using Filter Set 01 and Filter Set 20. Discrimination of early and late apoptotic cells was performed according to the previously described method [29,52]. Five different areas of each cell culture were analyzed. Each experimental group consisted of three cell cultures from different passages.

### 4.7. Extraction of RNA

Mag Jet RNA Kit (Thermo Fisher Scientific, Waltham, MA, USA) was used for the extraction of total RNA. The RNA quality was estimated by electrophoresis in the presence of 1 μg/mL ethidium bromide (2% agarose gel in Tris/Borate/EDTA buffer). The concentration of the extracted RNA was determined with NanoDrop 1000c spectrophotometer. RevertAid H Minus First Strand cDNA Synthesis Kit (Thermo Fisher Scientific, Waltham, MA, USA) was used for reverse transcription of total RNA.

### 4.8. Real-Time Polymerase Chain Reaction (RT-qPCR)

Each PCR was performed in a 25 μL mixture composed of 5 μL of qPCRmix-HS SYBR (Evrogen, Moscow, Russia), 1 μL (0.2 μM) of the primer solution, 17 μL water (RNase-free), 1 μL cDNA. Dtlite Real-Time PCR System (DNA-technology, Moscow, Russia) was used for amplification. Amplification process consisted of the initial 5 min denaturation at 95 °C, 40 cycles of 30 s denaturation at 95 °C, 20 s annealing at 60–62 °C, and 20 s extension step at 72 °C. The final extension was performed for 10 min at 72 °C. All the sequences were designed with FAST PCR 5.4 and NCBI Primer-BLAST software. The data were analyzed with Dtlite software (DNA-technology, Moscow, Russia). The expression of the studied genes was normalized to gene encoding Glyceraldehyde 3-phosphate dehydrogenase (GAPDH). Data were analyzed using Livak’s method.

### 4.9. Statistical Analysis

All presented data were obtained from at least three cell cultures from 2–3 different passages. All values are given as mean ± standard error (SEM) or as individual cellular signals in experiments. Statistical analyses were performed by paired t-test. Differences are significant * *p* < 0.05, ** *p* < 0.01, and *** *p* < 0.001. n/s—data not significant (*p* > 0.05). MS Excel, ImageJ, Origin 2016 (OriginLab, Northampton, MA, USA), and Prism GraphPad 7 (GraphPad Software, RRID: SCR_002798) software was used for data and statistical analysis.

## 5. Conclusions

The GABAergic neurons of the cerebral cortex are characterized by an increased level of expression of NCS-1, which performs a protective function in this type of neurons. It inhibits hyperexcitation during ischemia and hyperammonemia through the maintenance of the expression pattern of protective proteins, the calcium-binding protein parvalbumin, and the regulation of cytosolic calcium levels. The knockdown of NCS-1 in all types of neurons leads to changes in the expression of a number of genes responsible for the viability of neurons. This leads to an increase in the amplitude of the OGD-induced increase in [Ca^2+^]_i_, the induction of apoptosis and necrosis, the generation of spontaneous Ca^2+^-impulses, and the appearance of a global increase in [Ca^2+^]_i_ in GABAergic neurons during hyperammonemia. The obtained results demonstrate a high level of NSC-1 expression in GABAergic neurons and some neuroprotective mechanisms of this protein under pathological effects of ischemia/reoxygenation and hyperammonemia. The expression of NCS-1 primarily promotes the survival of GABAergic neurons, which in turn exert inhibition on the neuronal network and promote the survival of other neuron subtypes.

## Data Availability

The data presented in this study are available on request from the corresponding author.

## Supplementary Materials

The following supporting information can be downloaded at: https://www.mdpi.com/article/10.3390/ijms232415675/s1.
