# Supplementary material for: Neuronal Calcium Sensor-1 Protects Cortical Neurons from Hyperexcitation and Ca2+ Overload during Ischemia by Protecting the Population of GABAergic Neurons
Source: Int J Mol Sci. 2022 Dec 10;23(24):15675. doi: 10.3390/ijms232415675 (PMC9778989; doi:10.3390/ijms232415675)
Supplement: Supplementary file 1 [file ijms-23-15675-s001.zip › ijms-2031567-supplementary.pdf]

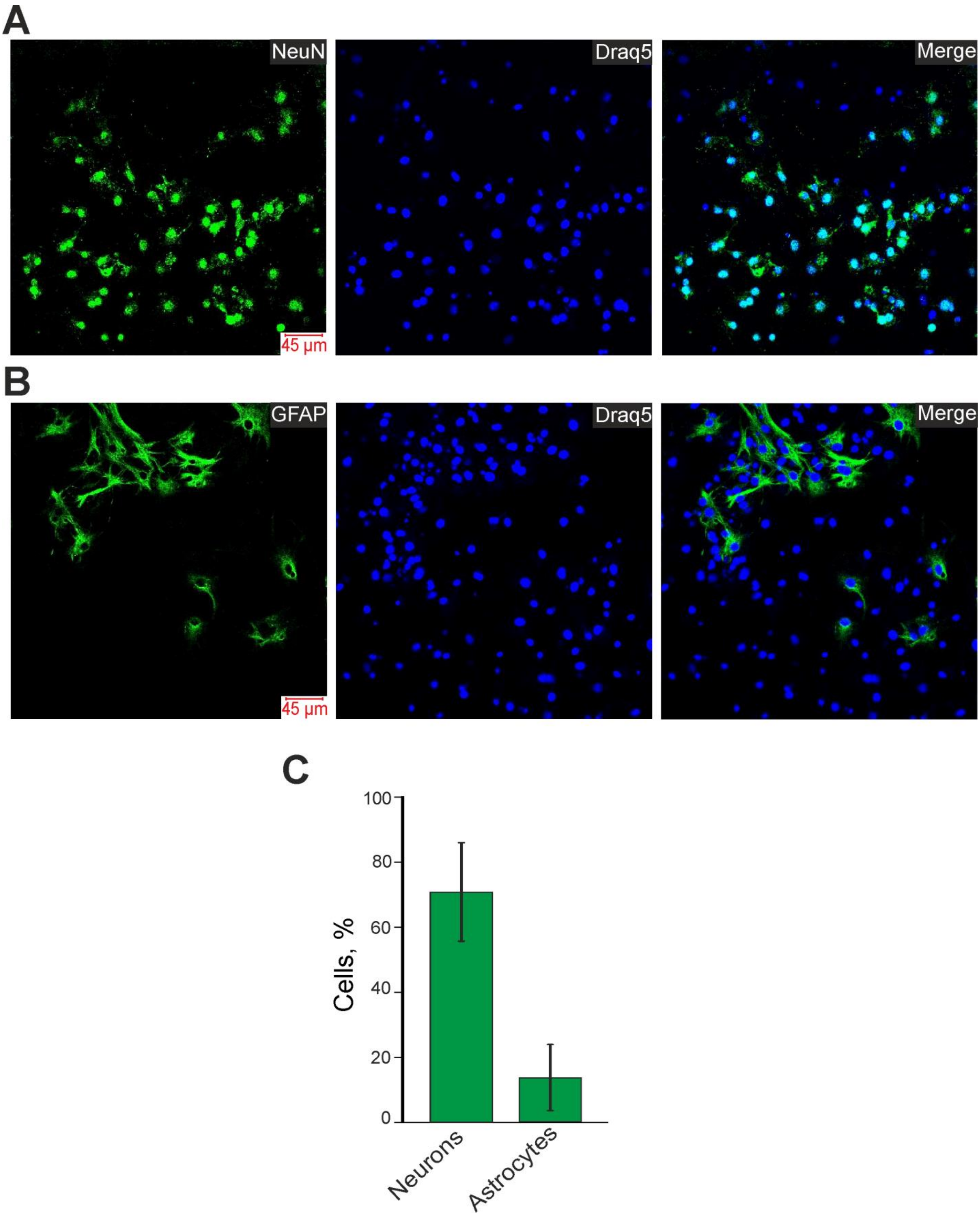

Supplementary Figure S1. Immunocytochemical staining of mouse cortical cells with antibodies against NeuN ((**A**), neuronal marker) and GFAP ((**B**), astrocytic marker). Cell nuclei were stained with Draq5. C - The number of neurons (NeuN positive cells) and astrocytes (GFAP positive cells) in cultured mouse cortical cells. The number of nuclei was taken as 100% (Draq5 stain).
